# Obstinate Dyspepsia Cured by the Use of Milk

**Published:** 1871-02

**Authors:** I. N. Danforth

**Affiliations:** Chicago


ARTICLE III.—*Obstinate Dyspepsia Cured by the use of Milk.* By I. N. DANFORTH, M.D., Chicago.

For the past few years, milk, as an article of diet, in certain morbid conditions, has been much lauded by numerous writers, and very frequently, as well as beneficially, employed both in private practice and in hospitals. It is rarely the case, however, that milk, or indeed any other exclusive article of diet, is so persistently adhered to, as in the instance which I propose to report at the present time.

Mr. N. W. Belknap, an intelligent gentleman, residing in Nokomis, Illinois, found himself, in the year 1853, suffering from an obstinate and very aggravated dyspepsia, which had harassed him for many years previous. He had been "doctored" with every conceivable remedy, without any result, save the merest temporary palliation of the more urgent symptoms. Constant gastralgia, obstinate constipation, progressive emaciation, sleeplessness and hypochondria, were the more prominent symptoms at the time alluded to. Discouraged and disheartened, he determined to take the case into his own hands. His first step was to "throw physic to the dogs," and abandon drugs entirely; in the next place, he commenced a careful study of his case, with the view of ascertaining what form of diet was most acceptable to his now exquisitely sensitive stomach. After a series of remarkably well conducted experiments, he found that fresh milk—"warm from the cow"—was most easily digested, and was therefore the proper article of diet for him to adopt. Accordingly, he commenced, at the date above specified (1853), the use of milk as his exclusive food. He was at this time twenty-four years old, and weighed 110 lbs., and, as I have already indicated, his condition was truly deplorable. Under the use of the milk diet, his health has been gradually restored until, at the present time, his weight is 170 lbs., and, in every regard, he presents the appearance of a robust man. He now consumes four quarts of fresh milk every twenty-four hours, together with about four ounces of stale bread. He eats no meat at all; occasionally he indulges slightly in vegetables, as turnips, cabbage, potatoes, and apples. A very remarkable feature of Mr. B.'s case is, that he drinks no water—"not a gallon of water since 1853"—but drinks milk instead. He always takes a pitcher of milk with him to his place of business, and makes it his constant beverage—not even drinking tea or coffee. I was much interested, also, in his statement that he felt little desire for any other kind of diet, but that he never tired of milk. It is a very common complaint among patients, when we attempt to restrain them to the exclusive use of milk, or, indeed, any other exclusive article of diet, that they soon tire of it, and in many instances it becomes so absolutely disgusting, that the stomach cannot be made to receive it, much less to retain it. Yet, here is a man who for *thirteen years* has made milk his all but exclusive diet, without even feeling it to be a hardship. An increase of body-weight might naturally have been looked for; but I think hardly any one would have anticipated so great a gain as 60 lbs. even in so long a time as this experiment has been in progress. One other point demands a passing notice: as a general thing, milk is likely to induce a tendency to constipation, so troublesome in some cases as utterly to preclude its use. Indeed, the "clogging" properties of milk are such a constant and not altogether unpardonable scare-crow to nurses, that it is frequently very difficult to get them to administer it in quantities sufficient to be of any great service. But in the present case, obstinate constipation has been replaced by almost clock-like regularity of the bowels. The discharges, Mr. B. informed me, are habitually light colored. Dr. S. Wier Mitchell, of Philadelphia, in a recent article in the *Medical Times*, states that "the stools begin to show the milk-tint—a yellowish or salmon hue—after forty-eight hours, and when the milk disagrees they are apt to be loose, while usually they are intensely tough and constipated." Dr. Mitchell's remarks relate to skimmed milk instead of fresh milk, however.

A microscopical examination of the urine in the case I have related, revealed nothing unusual, except what I had already anticipated, namely, the presence of a few free oil drops, although the proportion of oil was considerably less than I expected to find.

I have recorded this case at some length, because, in the first place, experiments in diet, conducted with such pains-taking persistency, are extremely rare, and when one does occur it is worth recording; and, in the second place, because it goes to confirm a conviction I have long entertained, that, when milk does not absolutely disagree with the stomach, we shall find in it our most efficient means of combating many cases of gastric disorder—and especially that distressing m ılady which is known as "acid dyspepsia."

In conclusion, I have to thank Mr. Belknap for his kindness in furnishing me with a history of his case, as well as for the permission he gave me to publish it ; and I may add that Dr. P. B. Cook, a cultivated and high-toned physician residing in Nokomis, assured me that the statements of Mr. B. were entirely reliable.

